# Account of Diseases in an Eastern District of London, from June, 20, to July 20, 1803

**Published:** 1803-08-01

**Authors:** 


### *Account of Diseases in an Eastern District of London, from June 20, to July 20, 1803.*

| | |
|---|---|
| **ACUTE DISEASES.** | |
| Typhus - - - - - - - | 8 |
| Hepatitis - - - - - - | 1 |
| Rheumatismus Acutus - | 3 |
| Cynanche Tonsillaris - | 2 |
| **CHRONIC DISEASES.** | |
| Tussis - - - - - - - | 10 |
| Tussis & Dyspnœa - - | 7 |
| Phthisis Pulmonalis - - | 3 |
| Hydrothorax - - - - | 2 |
| Anasarca - - - - - - | 3 |
| Gastrodynia - - - - | 4 |
| Enterodynia - - - - | 5 |
| Dyspepsia - - - - - | 6 |
| Obstipatio - - - - - - | 3 |
| Diarrhœa - - - - - - | 5 |
| Prolapsus Ani - - - | 1 |
| Hysteria - - - - - - | 3 |
| Hypochondriasis - - - | 2 |
| Herpes - - - - - - - | 7 |
| Rheumatismus Chronicus | 12 |
| **PUERPERAL DISEASES.** | |
| Peritonitis - - - - - - | 2 |
| Menorrhagia Lochialis - | 7 |
| Dolores Post Partum - | 4 |
| **INFANTILE DISEASES.** | |
| Apthæ - - - - - - - | 3 |
| Herpes - - - - - - - | 5 |
| Diarrhœa - - - - - - | 3 |
| Ophthalmia - - - - - | 2 |

